# Supplementary material for: LncAABR07053481 inhibits bone marrow mesenchymal stem cell apoptosis and promotes repair following steroid-induced avascular necrosis
Source: Commun Biol. 2023 Apr 3;6:365. doi: 10.1038/s42003-023-04661-0 (PMC10070412; doi:10.1038/s42003-023-04661-0)
Supplement: Supplementary file 2 — Supplementary Information [file 42003_2023_4661_MOESM2_ESM.pdf]

Supplementary Figures

Supplementary Figure 1: Isolation, culture, and identification of BMSCs.

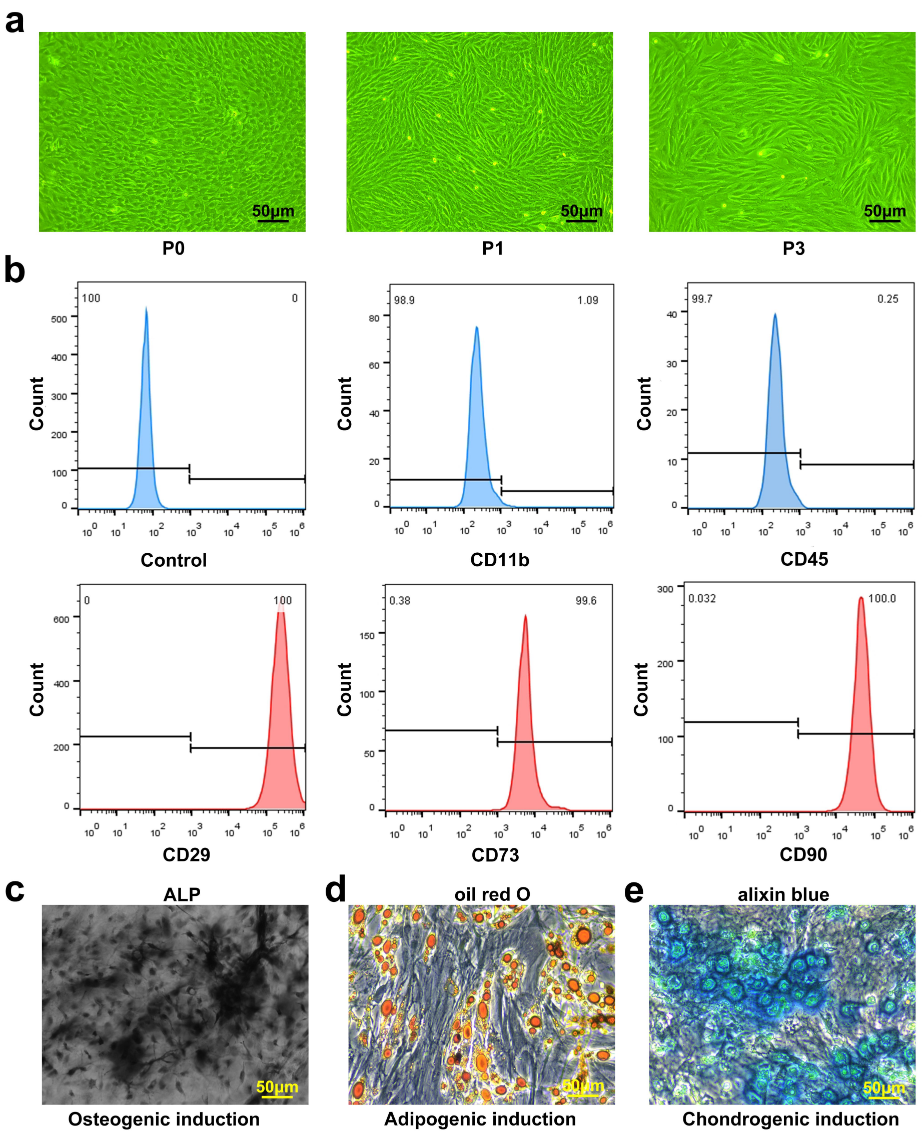

a. Morphological observation of BMSCs (n = 6). b. Flow cytometry analysis of the BMSC surface antigens CD11b, CD45, CD29, CD73, and CD90; the Control group is represented as a buffer negative control (n = 5). c. Observation of osteogenic differentiation of BMSCs by ALP staining (n = 5). d. Observation of adipogenic differentiation of BMSCs by oil red O staining (n = 5). e. Observation of chondrogenic differentiation of BMSCs by Alixin blue staining (n = 5).

Supplementary Figure 2: Effect of hypoxia on BMSCs.

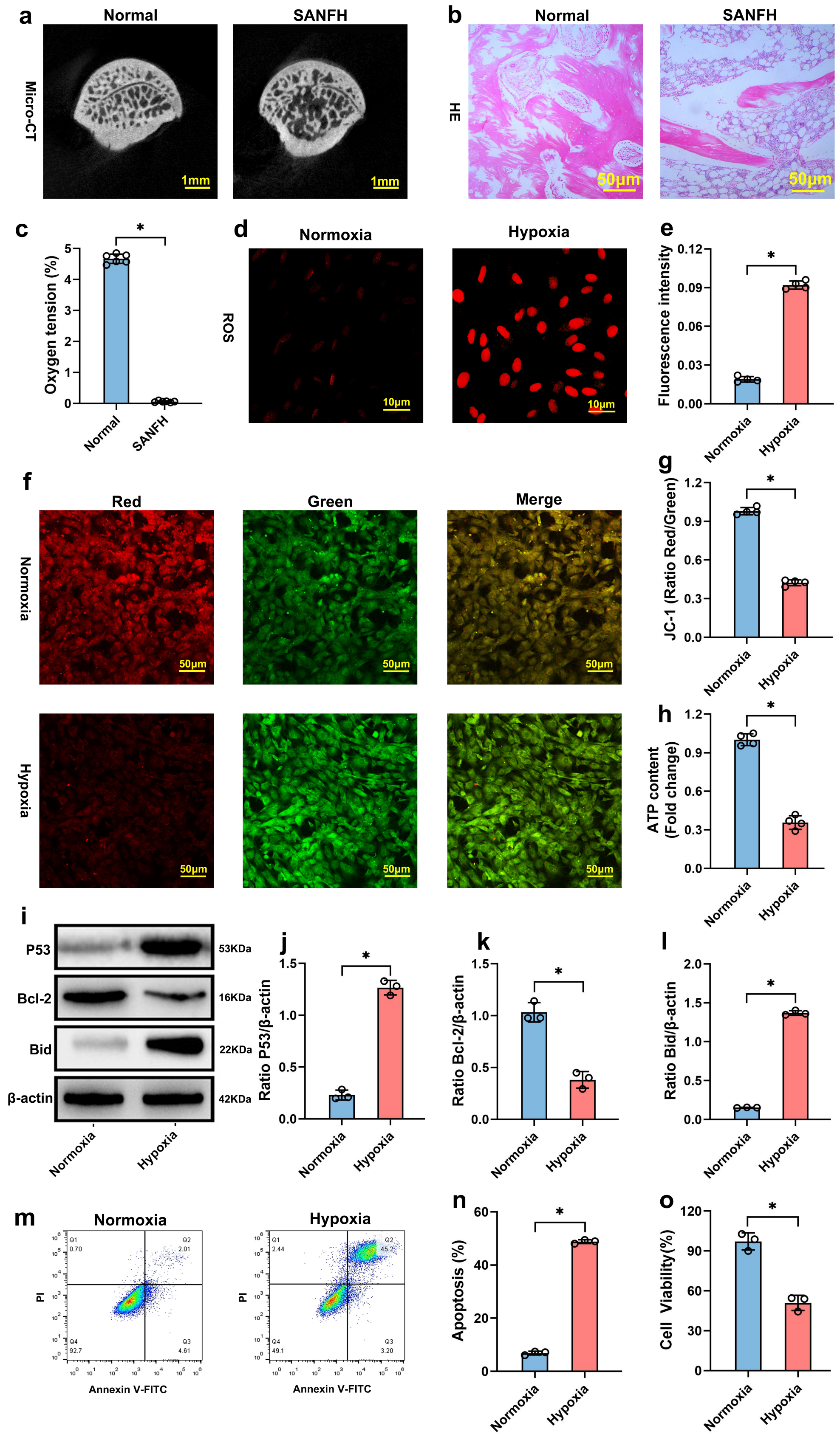

a-b. Evaluation of the SANFH model by micro-CT and H&E staining (n = 7). c.

Detection of the oxygen concentration in the area of osteonecrosis of the femoral head (n = 6). d–e. The content of reactive oxygen species (ROS) was detected by DHE (n = 4). f–g. Detection of mitochondrial membrane potential by JC-1 (n = 4). h. ATP content (n = 4). i–l. The expression levels of P53, Bcl-2, and Bid were detected by western blotting (n = 3). m–n. Detection of BMSC apoptosis under different conditions by Annexin V/PI (n = 3). o. Cell viability (n = 3). Data are shown as mean ± S.D. $^{*}P < 0.05$; In (c, e, g, h, j-l, n, o), statistical significance was calculated by Student's *t*-test.

Supplementary Figure 3: The uncropped images of the original western blots.

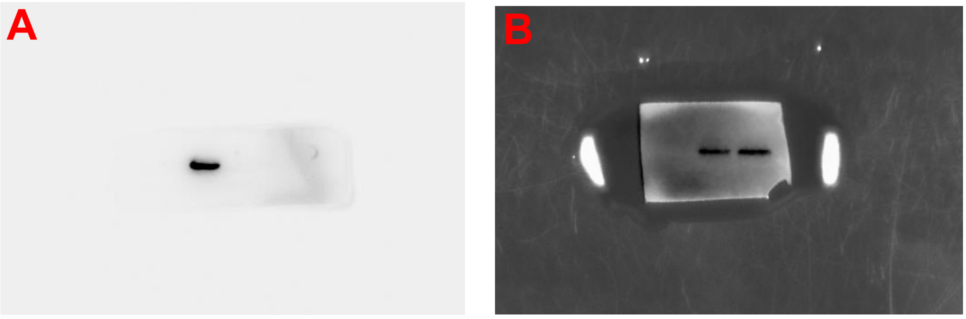

Supplementary figure from Figure 1D in the main manuscript: The expression of the Myc-fused protein. **(A)** Myc protein band detected by Western blots; **(B)** β-actin protein band detected by Western blots.

Grouped from left to right: ①pcNDA4-myc-KLF4, ②pcNDA4-myc-LncRNA.

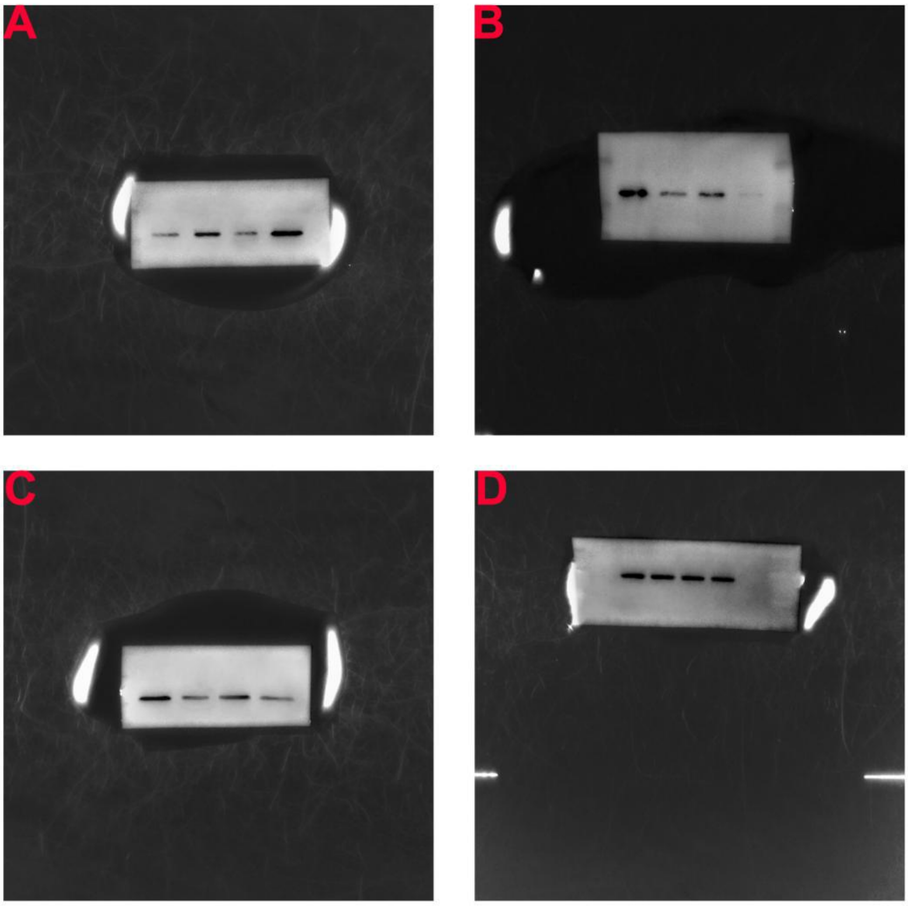

Supplementary figure from Figure 2F in the main article: Western blot analysis of the expression levels of Cleaved-CASP3, Survivin, and Bcl-2. **(A)** Cleaved-CASP3

protein band detected by Western blots; **(B)** Survivin protein band detected by Western blots; **(C)** Bcl-2 protein band detected by Western blots; **(D)** β-actin protein band detected by Western blots.

Grouped from left to right:

①BMSCs/Vec+normoxia, ②BMSCs/Vec+hypoxia,

③BMSCs/Oe-LncAABR07053481+hypoxia,

④BMSCs/Sg-LncAABR07053481+hypoxia.

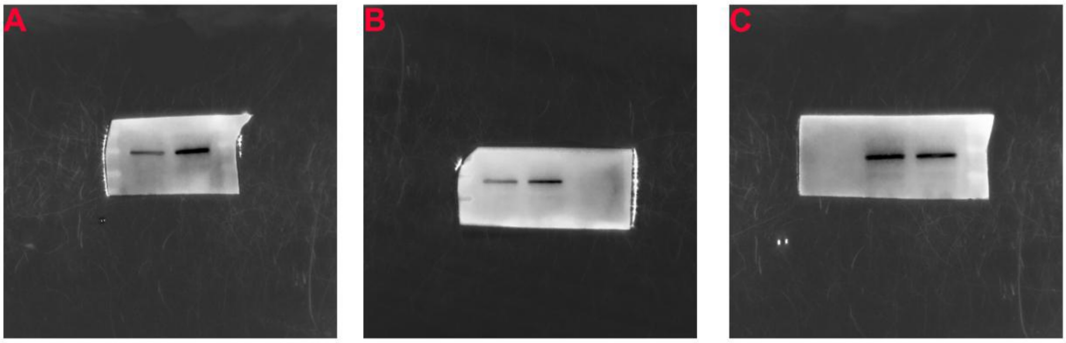

Supplementary figure from Figure 3E in the main article: The expression levels of Notch1 and NICD1 were detected by western blotting. **(A)** Notch1 protein band detected by Western blots; **(B)** NICD1 protein band detected by Western blots; **(C)** β-actin protein band detected by Western blots.

Grouped from left to right:

①BMSCs/Vec+hypoxia, ②BMSCs/Oe-LncAABR07053481+hypoxia.

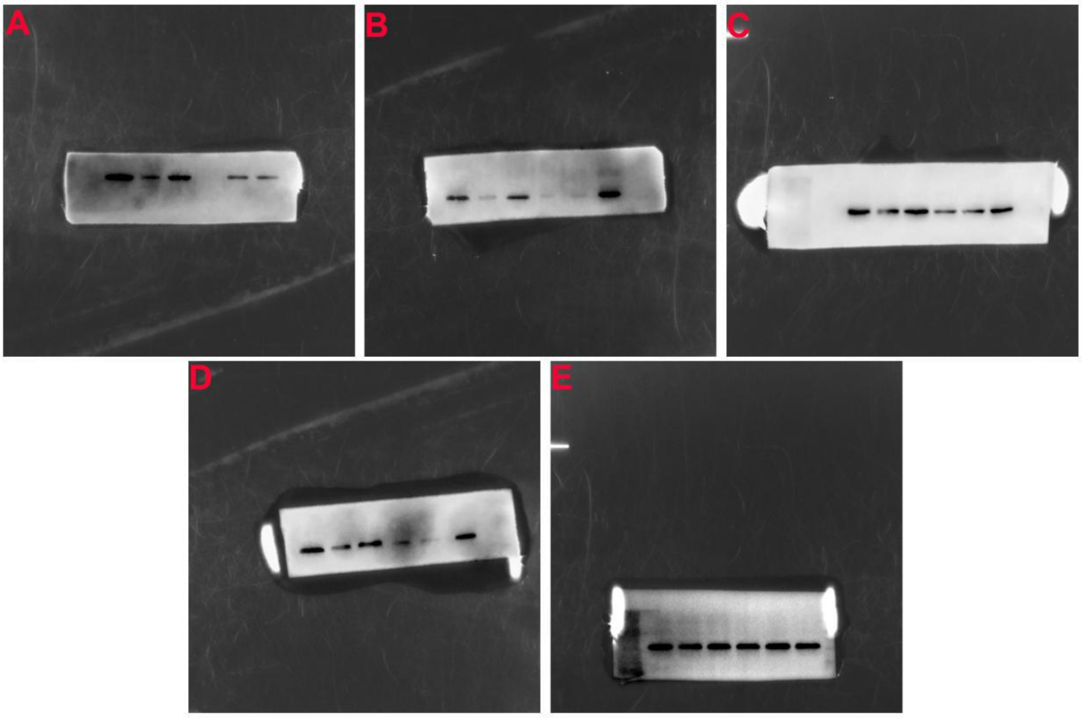

Supplementary figure from Figure 3J in the main article: The expression levels of Notch1, NICD1, Survivin, and Bcl-2 were detected by western blotting. **(A)** Notch1 protein band detected by Western blots; **(B)** NICD1 protein band detected by Western blots; **(C)** Survivin protein band detected by Western blots; **(D)** Bcl-2 protein band detected by Western blots; **(E)** β-actin protein band detected by Western blots.

Grouped from left to right:

① BMSCs/Vec+normoxia,

② BMSCs/Vec+hypoxia,

③ BMSCs/Oe-LncAABR07053481+hypoxia,

④ BMSCs/Oe-LncAABR07053481/Sg-Notch1+hypoxia,

⑤ BMSCs/Sg-LncAABR07053481+hypoxia,

⑥ BMSCs/Sg-LncAABR07053481/Oe-NICD1+hypoxia.

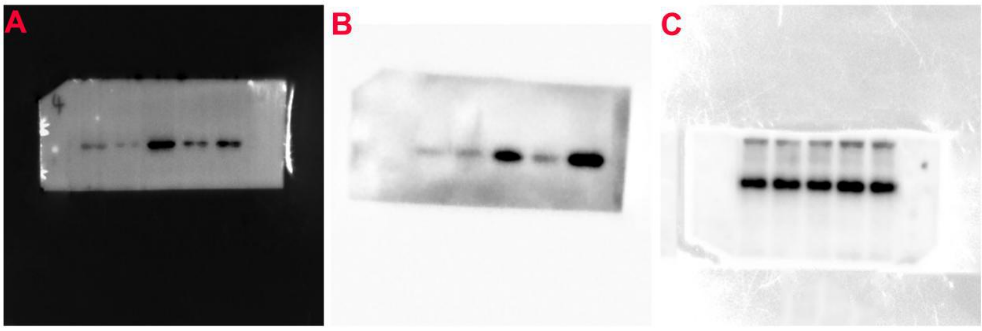

Supplementary figure from Figure 5D in the main article: The expression levels of Notch1 and NICD1 were detected by western blotting. **(A)** Notch1 protein band detected by Western blots; **(B)** NICD1 protein band detected by Western blots; **(C)** β-actin protein band detected by Western blots.

Grouped from left to right:

① BMSCs+hypoxia,

② BMSCs/Vec+hypoxia,

③ BMSCs/Oe-LncAABR07053481+hypoxia,

④ BMSCs/Oe-LncAABR07053481/Oe-miR-664-2-5p+hypoxia,

⑤ BMSCs/Oe-LncAABR07053481/Oe-miR-664-2-5p/Oe-NICD1+hypoxia.

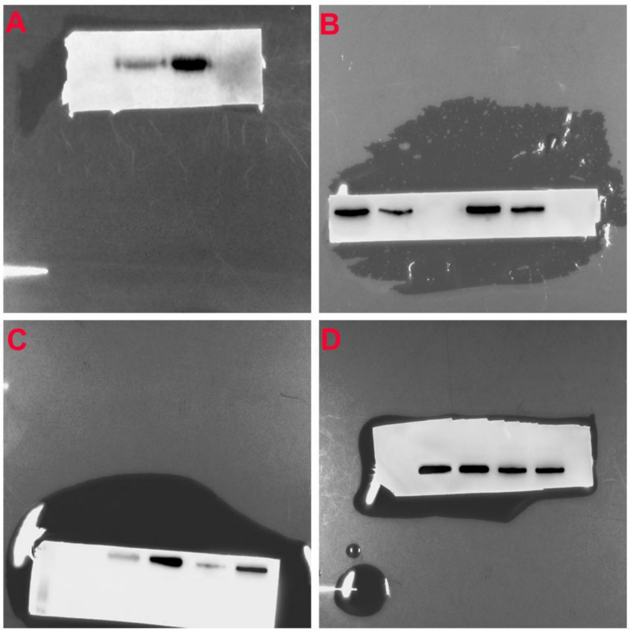

Supplementary figure from Supplementary Figure 2I in the main article: The expression levels of P53, Bcl-2, and Bid were detected by western blotting. **(A)** P53 protein band detected by Western blots; **(B)** Bcl-2 protein band detected by Western blots; **(C)** Bid protein band detected by Western blots; **(D)** β-actin protein band detected by Western blots.

Grouped from left to right:

①BMSCs+normoxia, ②BMSCs+hypoxia.
