# Supplementary material for: LncAABR07053481 inhibits bone marrow mesenchymal stem cell apoptosis and promotes repair following steroid-induced avascular necrosis
Source: Commun Biol. 2023 Apr 3;6:365. doi: 10.1038/s42003-023-04661-0 (PMC10070412; doi:10.1038/s42003-023-04661-0)
Supplement: Supplementary file 4 — Reporting Summary [file 42003_2023_4661_MOESM4_ESM.pdf]

nature portfolio

Corresponding author(s): DBPR COMMSBIO-22-1657B

Last updated by author(s): Feb 20, 2023

Reporting Summary

Nature Portfolio wishes to improve the reproducibility of the work that we publish. This form provides structure for consistency and transparency in reporting. For further information on Nature Portfolio policies, see our Editorial Policies and the Editorial Policy Checklist.

Statistics

For all statistical analyses, confirm that the following items are present in the figure legend, table legend, main text, or Methods section.

n/a	Confirmed	
☐	☒	The exact sample size (n) for each experimental group/condition, given as a discrete number and unit of measurement
☐	☒	A statement on whether measurements were taken from distinct samples or whether the same sample was measured repeatedly
☐	☒	The statistical test(s) used AND whether they are one- or two-sided<br>*Only common tests should be described solely by name; describe more complex techniques in the Methods section.*
☒	☐	A description of all covariates tested
☐	☒	A description of any assumptions or corrections, such as tests of normality and adjustment for multiple comparisons
☐	☒	A full description of the statistical parameters including central tendency (e.g. means) or other basic estimates (e.g. regression coefficient) AND variation (e.g. standard deviation) or associated estimates of uncertainty (e.g. confidence intervals)
☐	☒	For null hypothesis testing, the test statistic (e.g. F, t, r) with confidence intervals, effect sizes, degrees of freedom and P value noted<br>*Give P values as exact values whenever suitable.*
☒	☐	For Bayesian analysis, information on the choice of priors and Markov chain Monte Carlo settings
☒	☐	For hierarchical and complex designs, identification of the appropriate level for tests and full reporting of outcomes
☒	☐	Estimates of effect sizes (e.g. Cohen's d, Pearson's r), indicating how they were calculated

Our web collection on statistics for biologists contains articles on many of the points above.

Software and code

Policy information about availability of computer code

Data collection	The Applied Biosystems 7500 Fluorescent Quantitative PCR system; The GeneSpring GX v12.1 software package; Dual Luciferase reporter assay system; enzyme-labeling instrument; The OxygraphPlus software program; FlowJo_V10; Microsoft Excel.
Data analysis	SPSS version 20.0; GraphPad Prism version 7; The Agilent Feature Extraction software program 11.0.1.1; The web-based program RNAhydrid (https://bibiserv.cebitec.uni-bielefeld.de/rnahybrid/); The Gene Set Enrichment Analysis (GSEA) version 4.1.0; The Coding Potential Assessment Tool (CPAT); The NRecon image reconstruction software version 1.6; CTAn data analysis software version 1.9; CTVol 3D model visualization software version 2.0.

For manuscripts utilizing custom algorithms or software that are central to the research but not yet described in published literature, software must be made available to editors and reviewers. We strongly encourage code deposition in a community repository (e.g. GitHub). See the Nature Portfolio guidelines for submitting code & software for further information.

Data

Policy information about availability of data

All manuscripts must include a data availability statement. This statement should provide the following information, where applicable:

- Accession codes, unique identifiers, or web links for publicly available datasets
- A description of any restrictions on data availability
- For clinical datasets or third party data, please ensure that the statement adheres to our policy

The main data supporting the findings of this study are available within the manuscript and its Supplementary Information files. All other data are available from the corresponding author upon reasonable request.

Human research participants

Policy information about studies involving human research participants and Sex and Gender in Research.

Reporting on sex and gender	Research that does not involve humans
Population characteristics	Research that does not involve humans
Recruitment	Research that does not involve humans
Ethics oversight	Research that does not involve humans

Note that full information on the approval of the study protocol must also be provided in the manuscript.

Field-specific reporting

Please select the one below that is the best fit for your research. If you are not sure, read the appropriate sections before making your selection.

☒ Life sciences ☐ Behavioural & social sciences ☐ Ecological, evolutionary & environmental sciences

For a reference copy of the document with all sections, see nature.com/documents/nr-reporting-summary-flat.pdf

Life sciences study design

All studies must disclose on these points even when the disclosure is negative.

Sample size	For in vitro studies: n≥3 for gene expression and protein levels, microarray, fluorescence labeling of cells, luciferase activities, cell apoptosis. As for in vivo studies: n=6~7 for histological and bone morphometric analyses.
Data exclusions	No data were excluded from the analyses.
Replication	The data reported were generated using at least three different biological replicates in most required experiments. All experimental findings were reproduced for at least three times with similar results.
Randomization	Rats were allocated randomly.
Blinding	Each experiment was associated with proper controls, and compared samples were collected and analyzed under the same conditions.

Reporting for specific materials, systems and methods

We require information from authors about some types of materials, experimental systems and methods used in many studies. Here, indicate whether each material, system or method listed is relevant to your study. If you are not sure if a list item applies to your research, read the appropriate section before selecting a response.

Materials & experimental systems

n/a	Involved in the study	
☐	☒	Antibodies
☐	☒	Eukaryotic cell lines
☒	☐	Palaeontology and archaeology
☐	☒	Animals and other organisms
☒	☐	Clinical data
☒	☐	Dual use research of concern

Methods

n/a	Involved in the study	
☒	☐	ChIP-seq
☐	☒	Flow cytometry
☒	☐	MRI-based neuroimaging

Antibodies

Antibodies used	P53 (Abcam ab131442), Bcl-2 (Abcam ab196495), Bid (Abcam ab272880), Cleaved-CASP-3 (Abcam ab13847), Survivin (Abcam ab134170), myc (Abcam ab32072), β-actin (Abcam ab8227), IgG (Abcam ab6795), Notch1 (Cell Signaling Technology 3608), NICD1 (Cell Signaling Technology 4147), Anti-CD29/AF647, Anti-CD90/PE-CyTM7, Anti-CD73/PE, Anti-CD45/FITC, Anti-CD11b/V450 are purchased from BD.
Validation	All the primary antibody for the species and application statement on the manufacturer's websites.

Eukaryotic cell lines

Policy information about cell lines and Sex and Gender in Research

Cell line source(s)	bone marrow mesenchymal stem cells were obtained from young Sprague-Dawley (SD) rats (n = 18; body weight, 20–30 g),regardless of sex.
Authentication	Cells have been certified by surface antigens CD29, CD90, CD73, CD45 and CD11b
Mycoplasma contamination	The Cells used were tested to be negative for mycoplasma contaminations.
Commonly misidentified lines (See ICLAC register)	No commonly misidentified cell lines were used.

Animals and other research organisms

Policy information about studies involving animals; ARRIVE guidelines recommended for reporting animal research, and Sex and Gender in Research

Laboratory animals	The adult male Sprague-Dawley (SD) rats (n = 228; body weight, 500–600 g) were used to construct SANFH models.
Wild animals	The study did not involve wild animals.
Reporting on sex	No sex reports were involved in this study.
Field-collected samples	The study did not involve samples collected from the field.
Ethics oversight	The experiments were conducted in accordance with guidelines of the Experimental Animal Bioethics Committee of Guizhou Medical University.

Note that full information on the approval of the study protocol must also be provided in the manuscript.

Flow Cytometry

Plots

Confirm that:

☒ The axis labels state the marker and fluorochrome used (e.g. CD4-FITC).

☒ The axis scales are clearly visible. Include numbers along axes only for bottom left plot of group (a 'group' is an analysis of identical markers).

☒ All plots are contour plots with outliers or pseudocolor plots.

☒ A numerical value for number of cells or percentage (with statistics) is provided.

Methodology

Sample preparation	The proportion of mixed gas in the cell incubator (Thermo, USA) was adjusted to an oxygen concentration of 0%, nitrogen

Sample preparation	concentration of 95%, and carbon dioxide concentration of 5%. Then, BMSCs were placed in the mixed gas for continuous treatment for 48 h to induce hypoxia.
Instrument	flow cytometry (Beckman, USA).
Software	FlowJo_V10.
Cell population abundance	The cell purity for analysis was > 99%.
Gating strategy	The purity of the cells identified by surface antigen for analysis were all above 99%, and we selected more than 90% of the cell populations as the gating strategy and as the preliminary FSC/SSC gates of the starting cell population.

☒ Tick this box to confirm that a figure exemplifying the gating strategy is provided in the Supplementary Information.
